# Physiology

**Published:** 1857-01

**Authors:** 


### Bi-Monthly Periscope.

### PHYSIOLOGY.

1. Obliteration of the Portal Vein and the Secretion of Bile.—At the meeting of the French Academy of Sciences, Sept. 1, 1856, M. Oré presented a paper, detailing a series of experiments made with a view to ascertain the influence of obliteration of the portal vein upon the secretion of bile. The experiments were undertaken at the instance of M. Gintrac, of Bordeaux, who had observed, in his practice, a case which seemed to contradict the ordinarily received physiological doctrine, which teaches that the portal vein furnishes the liver with the materials for the secretion of bile.

The following are the author's conclusions:

"1. The secretion of bile having continued, after the partial and the complete obliteration of the portal trunk, I have concluded that it is not the blood of this vein which supplies the liver with material for its secretion. The gland is therefore dependent for its secretion upon the blood of the hepatic artery. The biliary secretion, like all others, is derived from the circulation of arterial blood. I have elsewhere established, that obliteration of the hepatic artery is not the proper means of testing the question, and cannot be considered, therefore, as weakening the conclusions which I am about to state.

"2. The secretion of sugar, by the liver, not having been altered by obliteration of the portal vein, is it not evident, as proved by M. Claude Bernard, that the production of saccharine matter is a proper secretion of the liver, and entirely independent of alimentation?

"3. The albuminous and glycogenous matters resulting from the digestion of feculent and albuminoid substances, being prevented from passing through the liver, are nevertheless not lost to the system, but enter the inferior cava vein by an anastomotic branch from the superior mesenteric vein.

"4. Finally, and it is with great hesitation that I put forth the statement, the arterial blood does not perform so important a part in the formation of sugar in the liver as in the secretion of bile."

After the reading of this paper, M. Andral stated, that in his medical practice he had observed a fact which fully substantiated the conclusions of M. Oré. A patient in whom there was reason to suspect, in consequence of external signs, an obliteration of the portal vein (a suspicion which was subsequently confirmed by an autopsical examination), not only did not present symptoms indicative of a suspension of biliary secretion, but yet gave evidence of a persistence of the glycogenous function by the occurrence of a diabetes.—*Revue Médicale, Oct.* 15, 1856. [Can it be possible that the distinguished medical savans of the French Academy, are ignorant of the fact, established by Kiernan and others, that the capillary terminations of the hepatic artery end in venous capillaries which open into the adjacent subdivisions of the portal vein, and are not, therefore, the radicles of the hepatic veins? A knowledge of this circumstance would, it seems to us, have furnished sufficient explanation of the continuance of the biliary secretion after obliteration of the portal trunk. But even if this were not the case, another fatal objection to the conclusions of MM. Oré and Andral is to be found in the fact that, when the portal vein is tied, its blood of course enters the general circulation, and in this way no small portion of it reaches the liver by the hepatic artery.—ED.]

2. THE TEMPERATURE OF THE BLOOD LOWERED BY PASSING THROUGH THE LUNGS.—At the sitting of same Academy, on the 15th of Sept., 1856, M. Claude Bernard read the continuation of his "Experimental Researches upon Animal Temperature," the second part of which relates to the modifications of temperature which the blood undergoes in traversing the respiratory organs. These experiments were made upon living animals, and M. Bernard thinks himself justified in coming to the following conclusions:

"1. That the circulation of the blood through the lungs causes a depression of the temperature of that fluid.

"2. That the lungs cannot therefore be considered a focus of animal heat.

"3. That the conversion of venous into arterial blood in the living animal, is not coincident with an augmentation in the temperature of that fluid, but, on the contrary, with a depression of its temperature."

In his next communication, M. Bernard proposes to examine into the modifications of temperature which the blood experiences in circulating through the genito-urinary apparatus.—*Ibid.*

[At a previous meeting of the Academy, M. Bernard, in a report of his experiments upon the modification of the temperature of the blood in its passage through the liver, established the fact that the blood is actually much warmer as it comes from that organ, than it is previous to its entrance.]

3. COAGULATION OF THE BLOOD.—In a recent letter to the London Lancet, Dr. Fred. W. Headland proposes a theory in regard to the coagulation of the blood, opposed to that advocated by Dr. B. W. Richardson, in his prize essay.

Sir:—When considering the subject of the chemical history of diabetes, in the year 1853, I was led, amongst other things, to conceive a certain theoretical explanation of the spontaneous coagulation of the blood. Having just now read over and reconsidered my notes taken at that time, I must say that I still prefer my theory to the one recently promulgated by Dr. B. W. Richardson, which it resembles in some points.

The statement, which I append below, is copied nearly verbatim from the notes referred to, but I may premise that the part of the idea which applies to *vegetable* physiology is the only part to which I have given any publicity, as at the time when it occurred to me I considered the whole matter of little moment.

The albuminous or nitrogenous matter found in the young growing cells of *a plant*, is in an insoluble condition. But on its passage in the fluids to those cells it must have been in some manner held dissolved in water. So, when the young cells get old and woody, they no longer contain this albuminous matter. On leaving them for the younger parts, it must again have assumed the soluble state. It seems to me that this phenomenon may be accounted for by supposing the albumen to be dissolved by an *alkaline* condition of the fluids, and deposited in some manner by the development of an *acid* at the particular point required.

The same may occur with the fibrine of animal blood, more prone to such coagulation than albumen. In the circulating blood of an animal, we find fibrine in the soluble state. In the tissues nourished by that blood it occurs in an insoluble form. But in the venous blood which carries away the effete products of those tissues, the fibrine is once more found in a state of solution. We are tempted to explain these facts in the same way as above: to suppose the fibrine held in solution by the presence of an alkali, and deposited in the insoluble form by the development of an acid. And, *in fact*, the blood is known to be feebly alkaline (from the presence of the carbonate or basic phosphate of soda), and the juice of muscle was found by Liebig to have an acid reaction.

But how is it that when the blood is withdrawn from the living body, its fibrine quickly assumes the solid or insoluble state—*i. e.* coagulates? By parity of reasoning, I suppose that this takes place again, by the development of an acid, to such an extent as simply to neutralize alkalinity, not so as to precipitate the albumen; this acid I believe to be *lactic acid.* It has been lately shown that glucose is always present in the living blood; and further proved, as I believe, that this glucose is continually changing into lactic acid, which, as fast as it forms, is bound or combined with oxygen to support the animal heat. When the blood is isolated, this vital process of combustion goes on no longer, lactic acid accumulates, the alkalinity of the natural blood is neutralized, and the fibrine coagulates. A tendency to nitrogenous decomposition may hasten this process, by supplying a ferment to act upon the glucose. Blood drawn in fevers coagulates quickly. Diabetic urine, allowed to stand for a time, may lose its sugar, and contain lactic acid. Extensive coagulation of the blood in the living body may be caused by injecting a putrefying liquid into the veins.

Another time I propose to comment at length, upon the theory of Dr. Richard son, and to state the motives which induce me to dissent from it. I am also planning a series of experiments, in order to put to a satisfactory test, the truth or falsity of the hypothesis sketched above.—*The Lancet.*

